# Two Letters with Answers

**Published:** 1881-01

**Authors:** 


WASHINGTON, PA., March 13, 1880.

THAD S. UP DE GRAFF, M. D.

*Dear Sir:*—Your favor received, in reply to your communication of the 9th, permit me to answer: Dr. F. Julius LeMoyne built a crematory for his family and any others of this community that wished to use it, and his idea was to bring about a reform in the present mode of disposing of the dead. His executors have permitted three bodies from a distance to be cremated in the furnace. We have over fifty letters from parties wishing to make arrangements incinerating their own bodies or those of their friends. We do not wish to commit ourselves to others, but if an urgent necessity seems to demand it, we might accommodate occasional parties that have not as yet written us, but prefer that other places should erect their own crematories, as we are not trying to make money out of the business, and indeed in every cremation have considerable of our time taken up. The cost of cremating a body is forty-five ($45) dollars after arriving in Washington.

We are 30 miles from Pittsburg and the same distance from Wheeling by rail. I send you herewith a copy of Dr. LeMoyne's arguments. Please send me a copy of your paper.

Yours truly,

V. HARDING, Executor
of DR. F. J. LEMOYNE.

This letter should have appeared in the June number of the BISTOURY, but was crowded out. We give it now for the information of those contemplating the building of cremation furnaces. It is to be hoped that people will take a more sensible view than now obtains, of disposing of their dead, and adopt the cleanly and wholesome method of incineration.

---

CHATHAM, NEW BRUNSWICK, July 22, '80.

THAD S. UP DE GRAFF, M. D.

*Dear Sir:*—I am laboring under a severe attack of "Camp Fever." The prodromic period has lasted six or seven years and the height of the fever has just been arrived at, or else (and I am rather inclined to this latter belief,) I was going through a mild form of the disease when I met a resident of Elmira, and an admirer of yours who placed in my hands a copy of "BODINES," and also of the "BISTOURY;" when, whew! the temperature went up to 106° in the shade, and I have been suffering from coma-vigil ever since, and some hint that I have been slightly delirious. I have completed a tent 12x12, a la Bodines, fly book, do; fishing coat, do; have four spaniels in training for the fall shooting, and have just completed a camp kit of pots, kettles, plates, teapot, cups, spoons, knives, sugar bowl, butter plate, tea canister, salt and pepper dishes, &c., &c., which all pack into a large galvanized pail 13½ inches high, by 14 inches in diameter. I have also invented a box for carrying out provisions to camp in sections, so that it can be taken apart when you get there and formed into a table and seats for four persons, thus doing away with one of the greatest inconveniences I used to experience in camping, viz: Eating your meals off the ground. I have been out fishing twice this year, but only for two days each time. The first time I caught 200, and the last time 400, but they were small, mostly about 1 pound, and the largest just turning 2 pounds. I intend taking one or two weeks shooting in the fall at the partridge and duck, and you may probably be inflicted with another scrawl about that time. I enclose $1.00 for the "BISTOURY." If it is half as good as "BODINES" the money could not be better invested.

Yours, Carphologically,

J. BAXTER, M. D.

If the doctor refers to trout—and he probably does—we should like to know what he terms big trout, when measured by the standard that those of 1 pound are small. To catch from 2 to 4 hundred pound trout in our mountain streams would be unheard of sport.

---

DENVER, COL., Nov. 20, 1880.

DR. UP DE GRAFF.

*Dear Sir:*—The BISTOURY has found its way out here, and to-day I took a run among my professional friends, exhibiting it to them. Here is the result: For the enclosed $6.00 send the BISTOURY for one year, commencing with the January number, to the following addresses:

D. B. J., M. D.

Thanks, Doctor. That is what might be termed a practical approval of our work. We would be glad to have others interest themselves in our behalf, and send us new names with their own. The information we give cannot be had for thrice the sum, elsewhere.

